# Population Based Trends in the Incidence of Hospital Admission for the Diagnosis of Hepatorenal Syndrome: 1998–2011

**DOI:** 10.1155/2016/8419719

**Published:** 2016-04-06

**Authors:** Manish Suneja, Fan Tang, Joseph E. Cavanaugh, Linnea A. Polgreen, Philip M. Polgreen

**Affiliations:** ^1^Department of Internal Medicine, University of Iowa Hospitals and Clinics, Iowa City, IA 52242, USA; ^2^Department of Biostatistics, University of Iowa, Iowa City, IA 52242, USA; ^3^Department of Pharmacy Practice and Science, University of Iowa, Iowa City, IA 52242, USA

## Abstract

*Background and Objectives*. Hepatorenal syndrome carries a high risk of mortality. Understanding the incidence and mortality trends in hepatorenal syndrome will help inform future studies regarding the safety and efficacy of potential therapeutic interventions.* Design and Methods*. We conducted a retrospective cohort study using the Nationwide Inpatient Sample. We identified hospitalizations from January 1998–June 2011 with a primary diagnosis of hepatorenal syndrome. To characterize the incidence trends in monthly hepatorenal syndrome hospitalizations, we fit a piecewise linear model with a change point at January 2008. We examined hospital and patient characteristics before and after the change point.* Results*. Hospital admissions with a diagnosis of hepatorenal syndrome increased markedly between September of 2007 and March of 2008. Comparing patients who were admitted with a diagnosis of hepatorenal syndrome prior to 2008 with those after 2008, we found that length of stay increased while the mortality of patients admitted for hepatorenal syndrome decreased.* Conclusion*. The revision of the diagnostic criteria for hepatorenal syndrome may have contributed to the increase in the incidence of admissions for hepatorenal syndrome. However, the changes in the principles of hepatorenal syndrome management may have also contributed to the increase in incidence and lower mortality.

## 1. Introduction

Hepatorenal syndrome (HRS) is a distinct form of functional kidney injury seen in end-stage liver disease. HRS is characterized by intense renal vasoconstriction in the setting of systemic and splanchnic arterial vasodilatation. The association between liver disease and renal failure had been known for more than a hundred years, but the first consensus definition of HRS was developed in 1994 by the International Ascites Club (IAC) with a new revised criterion introduced in 2006-07 [1]. HRS has a reported incidence of 10% among hospitalized patients with cirrhosis and ascites and is associated with a high mortality [1]. HRS is classified into 2 types: type-1 HRS is characterized by a rapid and often precipitous decline in renal function with a median survival of about 2 weeks, whereas with type-2 HRS, kidney failure occurs over a longer period of time, with a median survival of 6 months [2–5]. To date, reports on the incidence and mortality associated with HRS have mostly been based on single-center experiences, which may not reflect the nationwide incidence and burden of HRS-related hospital admissions.

A recent systematic review of HRS patients suggested an epidemiologic improvement in short-term mortality between 2005 and 2010 when compared to the 27 years between 1977 and 2004 [6]. Understanding the nationwide incidence and mortality trends in HRS will help hospitals plan for the resources needed to care for these patients and inform future studies regarding the safety and efficacy of potential interventions for the management of HRS.

The objective of this study was to determine how the rate of hospital admissions for HRS has changed over the past decade in the United States. To our knowledge, this is the first study that has examined the national incidence and mortality trends for hospital admissions with a diagnosis of HRS.

## 2. Methods

### 2.1. Data Source

We conducted a retrospective cohort study using the Nationwide Inpatient Sample (NIS). The NIS is the largest all-payer database of national discharges in the US. The database is maintained as part of the Healthcare Cost and Utilization Project by the Agency for Healthcare Research and Quality (AHRQ) and contains data from a 20% stratified sample of nonfederal acute care hospitals. To adjust for yearly changes in the sampling design, we applied the weights provided by AHRQ. All analyses were performed using R, version 2.15.1 (R Foundation for Statistical Computing).

We first identified all hospitalizations over the period from January 1998 through June 2011 during which a primary diagnosis of HRS was recorded. For HRS case ascertainment, we used the* International Classification of Diseases*,* 9th Revision*,* Clinical Modification (ICD-9-CM)* code 572.4. We then aggregated all cases by month to produce a national sample of cases of HRS over time. Cases were assigned to a calendar month on the basis of the date that the patient was admitted to the hospital. In a similar fashion, we compiled a sample of AKI- (acute kidney injury) cirrhosis cases. We identified all hospitalizations over the same time period during which a primary diagnosis of AKI was received and a secondary diagnosis of cirrhosis was also recorded, or a primary diagnosis of cirrhosis was received and a secondary diagnosis of AKI was then recorded. For AKI-cirrhosis case ascertainment, we used ICD-9-CM codes 574.5, 584.6, 584.7, 584.8, and 584.9 for AKI and 571.5, 571.2, and 571.6 for cirrhosis.

### 2.2. Statistical Analysis

We fit a piecewise (or segmented) linear regression model. A piecewise linear model is comprised of a series of linear models connected at “change points,” where shifts in the slope may occur. In our case, we determined a single change point for the HRS series by visual inspection. We detrended the series and conducted residual diagnostics to investigate whether there was any autocorrelation pattern in the residuals, as failure to account for such autocorrelation may lead to incorrect inferential conclusions. Temporal correlation in the residuals was examined by the autocorrelation function (ACF) and the partial autocorrelation function (PACF).

To examine if changes occurred in demographics and characteristics before and after the change point, the sample was divided into two groups based on the identified change point (based on the HRS series). For binary outcomes (e.g., gender, mortality) and categorical outcomes (e.g., race, hospital bed size), comparisons of proportions were conducted using the Pearson chi-square test. For continuous outcomes (e.g., length of stay and age), comparisons of means were conducted using Wilcoxon rank sum test.

## 3. Results

**Figure 1:**
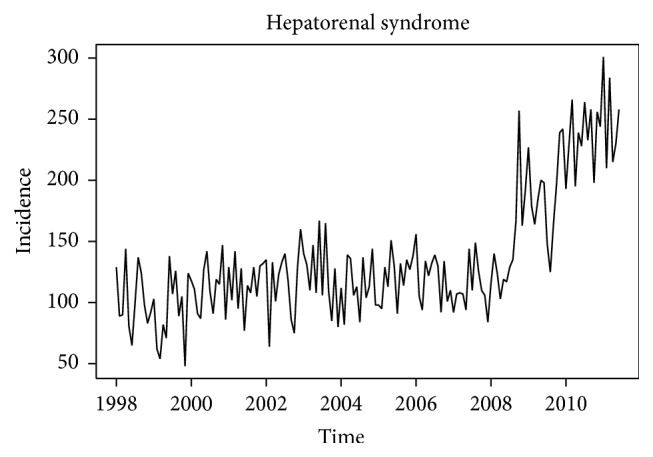
Monthly hospitalizations of patients diagnosed with hepatorenal syndrome in the United States (Nationwide Inpatient Sample, 1998–2011).

**Figure 2:**
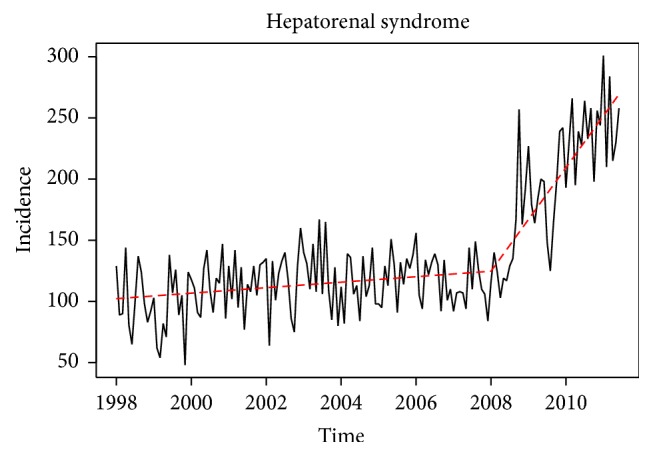
Fitted segmented linear regression model for hepatorenal syndrome incidence series based on monthly hospitalizations.

The overall time series plot of HRS incidence from 1998 to 2011 is shown in Figure 1. Based on a visual inspection of the plot, we chose January of 2008 to be the change point for our piecewise linear regression model. Upon inspection of the ACF and PACF for the residuals from the fitted model (Figure 2), we found no evidence of temporal correlation in the detrended HRS series, which implies that a segmented linear regression model based on independent errors is sufficient to characterize HRS incidence (data not shown).

Table 1 summarizes the model results for the HRS series. The yearly slope coefficient for the period before the change point (January of 2008) is positive and significant (*P* value = 0.0030) but small (slope estimate = 2.26; 95% CI: (0.81, 3.71)). In January of 2008, a substantial increase in the yearly slope is evident (change in slope estimate = 40.1; 95% CI: (33.84, 46.34); and *P* value < 0.0001).

Table 2 summarizes the means and proportions in the demographics and characteristics of HRS hospitalizations before and after January 2008. Specifically, mean age decreased after January 2008, and patients had a longer mean length of stay (*P* value < 0.0001). In addition, a significant decrease in the mortality rate was found with HRS patients after January 2008 (*P* value < 0.0001), and a higher proportion of patients received dialysis (*P* value < 0.0001). Finally, more patients were admitted to teaching and large hospitals after January 2008 (*P* value < 0.0001).

Table 3 summarizes the means and proportions in the demographics and characteristics of AKI-cirrhosis hospitalizations before and after January 2008 (i.e., based on the change point for the HRS series). Specifically, average age increased after January 2008 and patients had a shorter mean length of stay (*P* value < 0.0001). In addition, a significant decrease in the mortality rate was found with AKI-cirrhosis patients after January 2008 (*P* value < 0.0001). In contrast to the HRS cohort, patients had a lower probability of receiving dialysis after the change point (*P* value < 0.0001).

## 4. Discussion

Our results show that hospital admissions with a diagnosis of HRS increased markedly after January 2008. In addition, when we compared patients who were admitted with a diagnosis of HRS prior to January 2008 with those after January 2008, we found that length of stay increased over time. However, during the same period of time, the mortality of patients admitted for HRS decreased. Finally, the incidence of HRS patients treated with dialysis increased over this period of time.

Interestingly, multiple randomized controlled trials demonstrating the short-term efficacy of medical management of HRS were published in 2007-2008 [7–11]. Thus, it is possible that the publication of these studies was associated with a change in the management of HRS and perhaps improved outcomes. New recommendations included changing the choice of volume expander from saline to albumin. Albumin improves circulatory function in cirrhosis by expanding central blood volume and increasing cardiac output [12]. Some recent studies have shown that the administration of albumin to cirrhotic patients with SBP causes arterial vasoconstriction and blood pressure increase, probably attributable to the ability of albumin to bind to vasodilators [13]. A large body of evidence, based on observational studies and randomized controlled trials, has accumulated in the last decade showing that some of the new therapies represent a milestone in the management of HRS [14]. The demonstration that type-1 HRS can be improved by vasoconstrictors or norepinephrine alone or in conjunction with intravascular volume expansion with albumin and that reversal of type-1 HRS may be associated with improved survival represents a major change in our understanding of the syndrome. It is therefore conceivable that an improvement of renal function in patients with HRS treated with vasoconstrictors and albumin could be due to the additive effects that the compounds have on cardiac function and peripheral arterial circulation. Finally the use of high dose albumin in cases of HRS might have a favorable effect on effective circulating volume and thus improving clinical outcomes.

The change in the admissions for HRS and changes in outcomes could have been due to changes in practice management. Alternatively, the change we observed could also be in part due to the change in definition of HRS or inclusion criteria for HRS [4], which also occurred during the same period of time. The change in definition of HRS expanded the syndrome to include elements that were considered noninclusive in the older criteria; for example, an active infection is no longer an exclusion criterion. Finally, the increase in the incidence of HRS and a decrease in mortality in the HRS series could be due to a change in coding. However, we also analyzed the mortality rates of patients diagnosed with both AKI and cirrhosis (AKI-cirrhosis series). We found that the mortality rate for this series also decreased. Thus, it appears that outcomes have improved irrespective of the diagnostic codes after the change point.

Regardless of the cause of the increase in admissions for HRS, we anticipate a corresponding increase in healthcare resources used to treat this condition. If the treatment for HRS is truly more effective, it follows that it will be used more frequently, potentially leading to a dramatic increase in hospital charges related to the care for HRS. In addition to the number of admissions, we also noted that the length of stay associated with HRS admissions has increased during our study period and length of stay is one of the major drivers of healthcare costs [15]. Accurate estimates of the cost of HRS need to take into account readmissions and other posthospitalization events. Unfortunately, we cannot explore these issues using the NIS database. However, future HRS research should focus on readmissions and other outcome measures.

Our study has several limitations including the retrospective nature of the data. First, the NIS (similar to other administrative and hospital databases) is subject to coding errors and variability in coding illnesses. Also, NIS data does not allow for detailed individual chart reviews. Second, NIS data do not include medication records; thus we cannot investigate changes regarding specific therapeutics (e.g., national prevalence of albumin use). Third, as mentioned previously, we cannot follow patients following discharge. This is an important limitation to note because it limits our ability to determine if patients with HRS received liver transplants during subsequent hospitalizations during our study period. Furthermore, although the admissions for HRS increased, we are not able to determine how frequently readmissions occur nor can we attribute the increased volume of admissions to one-time versus multiple admissions. Finally lack of availability of specific therapies (e.g., terlipressin) in United States makes our findings less generalizable in countries where these therapies are available.

Despite the limitations of the study, we were able to explore the incidence and rate of hospitalizations for the diagnosis of HRS on a population level across the United States, and our results will help inform future studies both regarding resource use and longer-term studies of the efficacy of new approaches to HRS treatments. Our results also highlight the need for future studies to estimate the attributable cost of HRS.

## 5. Conclusions

The national trend in management of HRS shows that it continues to be a significant source of morbidity and mortality in patients with end-stage liver disease in the United States. The revision and broadening of the diagnostic criteria may have partially contributed to the increase in the incidence of admissions for HRS. Although we are unable to conclusively comment on the decrease in the overall mortality of HRS since 2008, we speculate that the widespread changes in the principles of management, including the use of volume expansion and vasopressors, may have played a part. The HRS patient cohort deserves further prospective examination in the near future.

## Figures and Tables

**Table 1:** Results of the fitted segmented linear regression model, characterizing the change in the yearly slope after 2008.

Diagnosis	Parameter	Estimate	Std. error	*t*-value	*P* value
HRS	Slope before change point	2.26	0.74	3.02	0.0030
	Change of slope	40.09	3.19	12.56	<0.0001^*∗*^

^*∗*^Note that because the change point was identified based on visual inspection, this *P* value must be interpreted liberally, since it is based on a post hoc test.

**Table 2:** Demographics and characteristics of patients diagnosed with HRS during hospitalization.

	Before the change point	After the change point	*P* value
Age, mean (SD), years	59.26 (13.99)	58.00 (12.22)	0.0048
Length of stay, mean (SD), days	7.74 (9.19)	8.91 (11.37)	<0.0001
Race, *n* (%)			0.1480
White	1594 (71.38)	1163 (72.64)	
Black	245 (10.97)	155 (9.68)	
Hispanic	275 (12.32)	180 (11.24)	
Asian or Pacific Islander	45 (2.02)	28 (1.75)	
Native American	21 (0.94)	26 (1.62)	
Other	53 (2.37)	49 (3.06)	
Mortality, *n* (%)			<0.0001
Alive	1615 (53.89)	1246 (67.98)	
Died	1382 (46.11)	587 (32.02)	
Gender, *n* (%)			0.6347
Male	1911 (63.59)	1180 (64.27)	
Female	1094 (36.41)	656 (35.73)	
Hospital teaching status, *n* (%)			<0.0001
Nonteaching	1677 (56.24)	878 (48.35)	
Teaching	1305 (43.76)	938 (51.65)	
Hospital bed size			<0.0001
Small	446 (14.96)	207 (11.40)	
Medium	856 (28.71)	438 (24.12)	
Large	1680 (56.34)	1171 (64.48)	
Procedures, *n* (%)			<0.0001
Dialysis	391 (13.01)	405 (22.06)	
Primary payer, *n* (%)			0.7733
Medicare	1198 (39.92)	706 (38.54)	
Medicaid	526 (17.53)	339 (18.50)	
Private insurance	917 (30.56)	557 (30.40)	
Self-pay	221 (7.36)	134 (7.31)	
No charge	20 (0.67)	17 (0.93)	
Other	119 (3.97)	79 (4.31)	

**Table 3:** Demographics and characteristics of patients diagnosed with AKI-cirrhosis during hospitalization.

	Before the change point	After the change point	*P* value
Age, mean (SD), years	58.83 (13.03)	60.00 (12.47)	<0.0001
Length of stay, mean (SD), days	11.02 (12.91)	9.30 (11.36)	<0.0001
Race, *n* (%)			0.0335
White	13564 (65.14)	9555 (65.57)	
Black	2507 (12.04)	1685 (11.56)	
Hispanic	3634 (17.45)	2489 (17.08)	
Asian or Pacific Islander	419 (2.01)	272 (1.87)	
Native American	185 (0.89)	138 (0.95)	
Other	513 (2.46)	433 (2.97)	
Mortality, *n* (%)			<0.0001
Alive	19115 (70.98)	14047 (84.43)	
Died	7816 (29.02)	2590 (15.57)	
Gender, *n* (%)			0.8224
Male	17256 (64.00)	10679 (64.11)	
Female	9706 (36.00)	5979 (35.89)	
Hospital teaching status, *n* (%)			0.2657
Nonteaching	11117 (41.50)	6732 (40.95)	
Teaching	15673 (58.50)	9706 (59.05)	
Hospital bed size			<0.0001
Small	2349 (8.77)	1324 (8.05)	
Medium	6552 (24.46)	3538 (21.52)	
Large	17889 (66.77)	11576 (70.42)	
Procedures, *n* (%)			<0.0001
Dialysis	4017 (14.90)	1922 (11.54)	
Primary payer, *n* (%)			<0.0001
Medicare	11104 (41.31)	7158 (43.09)	
Medicaid	5394 (20.07)	3418 (20.58)	
Private insurance	7513 (27.95)	4230 (25.47)	
Self-pay	1702 (6.33)	1050 (6.32)	
No charge	198 (0.74)	133 (0.80)	
Other	967 (3.60)	622 (3.74)	
